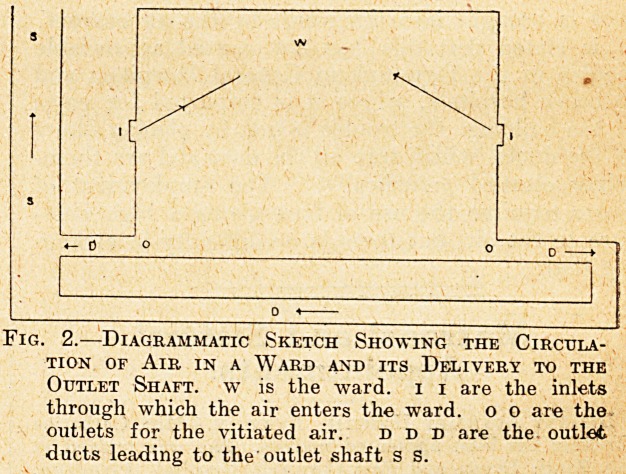# The Birmingham General Hospital

**Published:** 1916-09-23

**Authors:** 


### THE HEATING OF HOSPITALS.

### IX. The Birmingham General Hospital.

### THE "PLENUM" SYSTEM.

IN the "Plenum" system, as arranged at Birmingham, the whole of the heating and ventilating is done by the air circulating through the building. Natural ventilation as usually understood, the ventilation of the open window, is rigidly barred, and there are no fireplaces or chimneys. The windows do not open. The air for the building is taken down a shaft outside the building and through a hole in the wall, in which a fan is so placed that all the air is obliged to pass through it. The cleaning apparatus is fixed at the bottom of the shaft outside the building. It consists of a kaiar screen kept thoroughly wetted by the aid of a number of small streams of water issuing from a perforated pipe fixed above it. In addition, at certain intervals a flush of water is discharged over the screen from above, very much as in public lavatories, the object being to dislodge any substances that may have been caught in the meshes of the

FIG. 1.—DIAGRAMMATIC SKETCH SHOWING THE ARRANGEMENT FOR CLEANING, WARMING, AND DISTRIBUTING THE AIR OF THE WARDS UNDER THE "PLENUM" SYSTEM AT THE BIRMINGHAM GENERAL HOSPITAL. S S is the shaft through which the air enters the building. D D D D D D are the ducts through which the air is distributed to the different parts of the building. K is the kaiar screen. F F F are the fans delivering the air to the ducts. G G G are the grids of steam pipes arranged to warm the air.

screen. When the plant is overhauled, which is done periodically, the screen is subjected to a further cleansing by means of water under pressure played over it from the nozzle of a fire hosepipe.

In passing through the screen the air is deprived of all foreign matter, such as finely divided carbon and the vapours issuing from the different manufactories. The air is cooled to a few degrees above the temperature of the cleansing water; so that it is quite practicable to lower the temperature of the air to any extent that may be desired by employing cleansing water of a few degrees below that figure. The whole of the air entering the building is necessarily at one temperature, but a higher temperature may be given to the air in any part of the building by properly arranging the heating plant for that part. There is a grid of steam pipes in the neighbourhood of the screen to prevent the freezing of the cleansing water in very cold weather.

Within the building the air is distributed by fans to the different blocks through ducts of decreasing area, shown diagrammatically in Fig. 1. The large size of the main duct allows the air to be driven through it with a small pressure. From the main duct smaller ducts lead to different portions of the building. Near the entrance of each branch duct grids of vertical steam pipes are arranged; the air entering the duct passes over them, and any temperature that is required can be imparted to it. Steam traps are fitted to the steam grids; water formed by the condensation of the steam is drained off by these to the tank from which the boiler is fed.

The temperature in the wards, corridors, etc., is maintained at 62° F. day and night, winter and summer. The air is led in by inlet ducts arranged in various ways, but always delivering the air into the room at about eight feet above the floor line. The outlets are all fixed near the floor line; they are the same in number as the inlets, but are slightly larger. Considerable ingenuity has been shown by the architect in concealing the entrances to the ducts, to prevent any unsightly appearance. In the wards,

FIG. 2.—DIAGRAMMATIC SKETCH SHOWING THE CIRCULATION OF AIR IN A WARD AND ITS DELIVERY TO THE OUTLET SHAFT. W is the ward. I I are the inlets through which the air enters the ward. O O are the outlets for the vitiated air. D D D are the outlet ducts leading to the outlet shaft S S.

for instance, the inlet ducts form part of the lower window sills; the air passes up through them into the ward in a nearly vertical direction, and curves over at about the height mentioned above, eight feet above the floor line. The outlet ducts are placed under the beds. There are two distinct sets of air currents flowing into each ward, from opposite sides, and flowing out again each on its own side. All the outlet ducts are connected to another shaft, practically a chimney, in a part of the building some distance from the inlet shaft. Fig. 2 shows the arrangement of the outlet ducts and shaft. The air passing out of the building is usually lighter than that entering, which assists the circulation of the air through the building.

There are said to be no draughts anywhere. Entrance to the building is by swing doors, which are fairly frequently on the move, but the opening or closing of one of them appears to make no difference to the atmosphere inside the building. There is absolutely no sense of draught, nor any feeling of change in the air when passing from a corridor into a ward, or *vice versa.* The air current also performs the very important service of carrying off all unpleasant smells. During meal times there is absolutely no smell of any kind. At "screen time" the unpleasant smells that are sometimes present are completely carried off by the air current.

Disadvantages.

It is some years since the Birmingham General Hospital was fitted with the "Plenum" system, but no large new hospital has recently employed it. Word comes from America also that it is falling into disrepute there; several hospitals which adopted it originally have shut down the "Plenum" apparatus and adopted other methods, owing to the cost of maintenance and the space occupied by the ducts.

Doctors and nurses complained of the effect upon themselves of sleeping in "Plenum" air; that they got up in the morning feeling stale. At Birmingham, the writer understands, the "Plenum" system was cut off from the doctors' and nurses' quarters after a little time, and at the Belfast Victoria Hospital the doctors' and nurses' quarters were specially excluded from the system when the building was designed.

Another complaint that has been made is that dust is brought into the wards by the air current. It is stated that this has not caused appreciable trouble in the Birmingham General Hospital; and in other hospitals, where it is employed for parts of the building, the trouble is met by placing filters in the ducts, which trap all the dust and are themselves cleaned periodically. The employment of filters requires the use of fans giving a high pressure to the air, but there is no difficulty in arranging that.

## Figures and Tables

**Fig. 1. f1:**
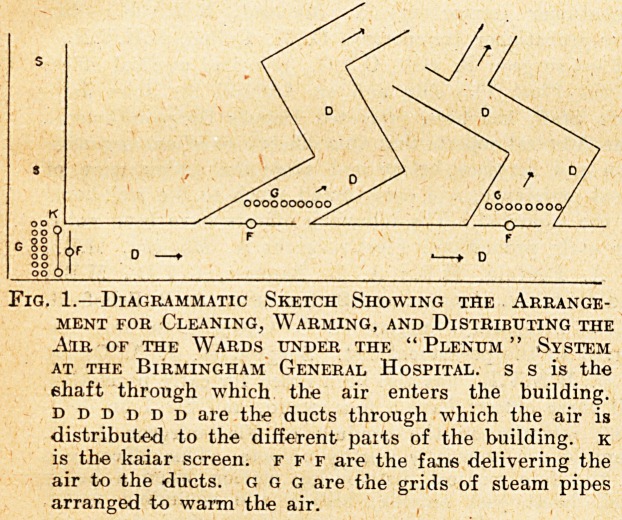


**Fig. 2. f2:**